# A simple score-based strategy to improve equity of the UK biennial diabetic eye screening protocol among people deemed as low risk

**DOI:** 10.1007/s00125-025-06379-6

**Published:** 2025-03-12

**Authors:** Matilda Pitt, Abraham Olvera-Barrios, John Anderson, Louis Bolter, Ryan Chambers, Alasdair N. Warwick, Samantha Mann, Laura Webster, Jiri Fajtl, Sarah A. Barman, Catherine Egan, Adnan Tufail, Alicja R. Rudnicka, Christopher G. Owen, on behalf of the ARIAS Research Group

**Affiliations:** 1 Population Health Research Institute, St George’s School of Health and Medical Sciences, City St George’s, University of London, London, UK; 2 Moorfields Eye Hospital NHS Foundation Trust, London, UK; 3 NIHR Biomedical Research Centre at Moorfields Eye Hospital NHS Foundation Trust and UCL Institute of Ophthalmology, London, UK; 4 Homerton Healthcare NHS Foundation Trust, London, UK; 5 South East London Diabetic Eye Screening Programme, Guy’s and St Thomas’ NHS Foundation Trust, London, UK; 6 Department of Ophthalmology, Guy’s and St Thomas’ NHS Foundation Trust, London, UK; 7 School of Computer Science and Mathematics, Kingston University, London, UK

**Keywords:** Diabetic eye screening, Equitable performance, Low risk, Screening frequency

## Abstract

**Aims/hypothesis:**

Biennial, as opposed to annual, screening for diabetic retinopathy was recently introduced within England for those considered to be at ‘low risk’. This study aims to examine the impact that annual vs biennial screening has on equitable risk of diagnosis of sight-threatening diabetic retinopathy (STDR) among people at ‘low risk’ and to develop an amelioration protocol.

**Methods:**

In the North East London Diabetic Eye Screening Programme (NELDESP), 105,083 people without diabetic retinopathy were identified on two consecutive screening visits between January 2012 and September 2023. Data for these individuals were linked to electronic health records (EHR). Characteristics associated with subsequent STDR diagnosis were identified (including age, gender, ethnicity and diabetes duration), and logistic regression was performed to identify people who require annual screening, using variables available to the NELDESP and data from EHR. Simulations of the biennial screening protocol, and of protocols incorporating the outcomes of the logistic models and a simplified points model, were implemented, and the relative risk of STDR calculated at each screening appointment was compared amongst various population subgroups. The results were validated using data from the South East London DESP.

**Results:**

Among the low-risk participants, there were 3694 incident STDR cases over a mean duration of 5.0 years (SD 3.4 years). Under the biennial screening protocol, almost all groups had a significantly higher risk of STDR diagnosis compared with people aged 41 years or older who were of white ethnicity and had been living with diabetes for <10 years. Compared with biennial screening, a simplified screening protocol based on age, diabetes duration and ethnicity reduced the number of delayed STDR diagnoses from 39% to 25%, with a more equitable performance across population groups, and a modest impact on screening appointment numbers (46% vs 57% reduction in annual screening appointments, respectively).

**Conclusions/interpretation:**

A simple, clinically deliverable, personalised protocol for identifying who should be screened annually or biennially for diabetic eye disease would improve equity in risk of delayed STDR diagnosis per appointment.

**Graphical Abstract:**

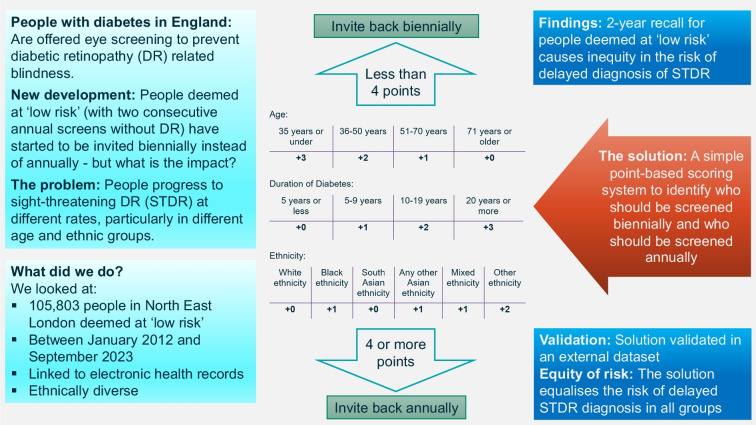

**Supplementary Information:**

The online version of this article (10.1007/s00125-025-06379-6) contains peer-reviewed but unedited supplementary material.


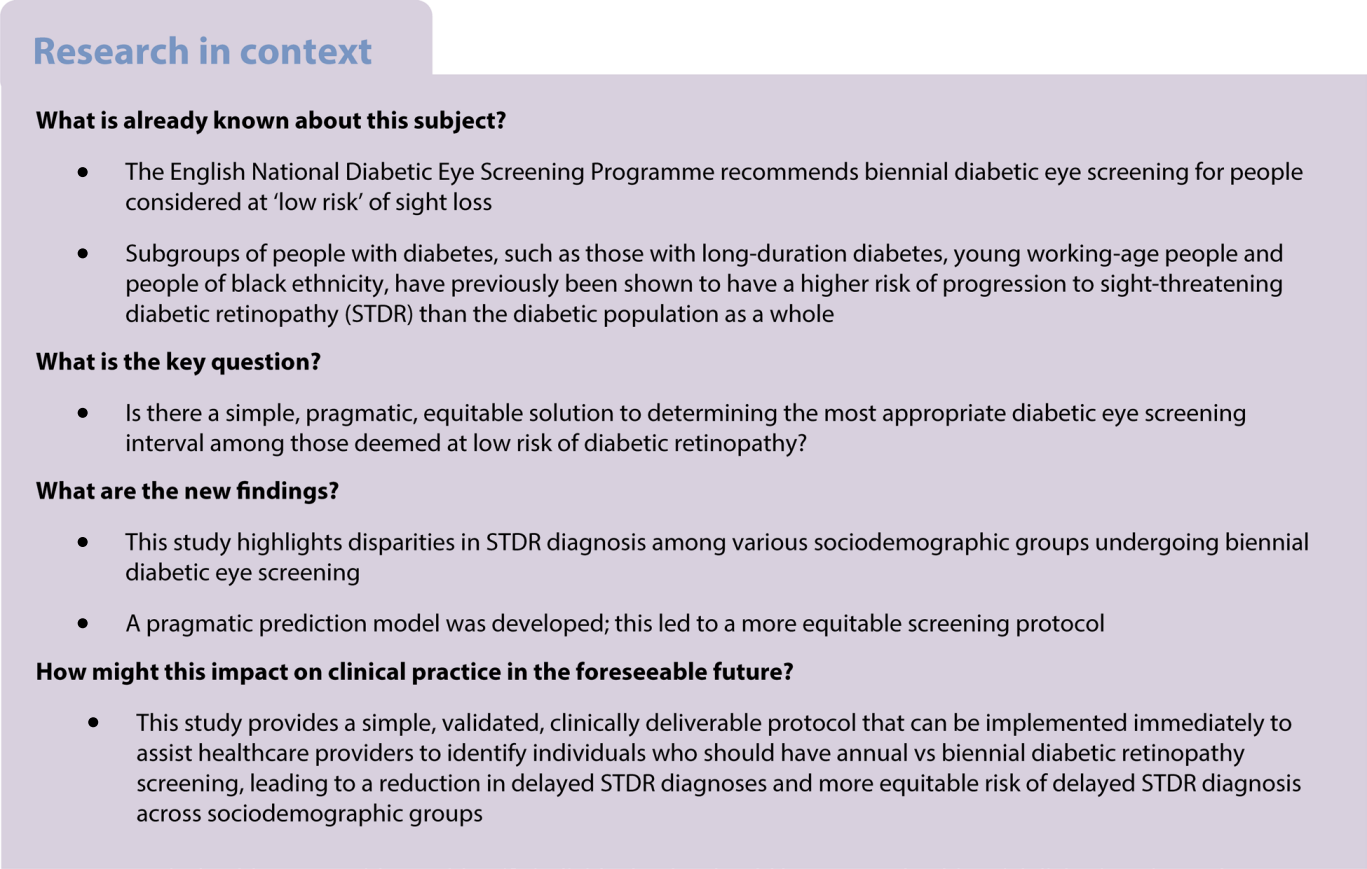


## Introduction

Diabetic retinopathy is a common complication of diabetes that may result in sight loss [1, 2]. Early treatment of sight-threatening diabetic retinopathy (STDR) can prevent or delay sight loss, and hence early diagnosis is key [3]. For this reason, since 2003, all people with diabetes aged over 12 years in the UK have been offered annual eye screening as part of the NHS Diabetic Eye Screening Programme (DESP) [4]. The number of people with diabetes is rising [5, 6] and, thus, there is increasing and potentially unsustainable pressure on diabetic eye screening services. A suggestion to alleviate pressure on screening services and reduce costs is to reduce the number of appointments per patient by increasing the time between appointments for people deemed at low STDR risk [7]. Low-risk individuals can be identified by using individualised risk predictions of STDR [8] or by a simpler method based on results from previous screening appointments [9].

Whereas people living with diabetes were previously offered appointments at least every year, guidelines were recently introduced meaning that, since October 2023, people without detectable diabetic retinopathy on two consecutive screening visits (i.e. those considered to be at ‘low risk’) are offered appointments every 2 years, i.e. biennially [10]. While some previous research has suggested that it would be safe and effective to extend the time period between screening appointments for these ‘low-risk’ people [11–14], this research has mainly included people of white ethnicity. In contrast, a systematic review suggested that there was insufficient evidence to warrant extending the time period between screening appointments [15], with recent research suggesting that extending the time period may delay STDR detection, especially among certain sociodemographic groups [16].

Risk factors for progression of diabetic retinopathy include duration of diabetes, diabetes type, suboptimal control of diabetes, ethnicity and higher BP [17–20]. There has been previous research on developing and assessing individualised risk scores that may be used to vary the interval between screening appointment times with promising results; however, use of such scores has yet to be implemented on a larger scale, potentially due to their complicated formulae and the need for access to medical records [8, 21–24].

We used data to develop simple risk prediction models for people with two successive negative diabetic eye screens, who were deemed at ‘low risk’ of progression to STDR, to identify who should attend diabetic eye screening annually and who should attend biennially, to enable a more equitable spread of the risk of STDR diagnosis at the time of the scheduled screening appointment. We used data that are routinely available in the North East London DESP (NELDESP) and additional data from linked electronic health records (EHR) to create various risk prediction models. We then sought to simplify the optimal model into a points-based protocol, with replication in a different dataset (the South East London DESP [SELDESP]). Crucially, the multi-ethnic nature of the datasets used allowed us to quantify the performance of these models in reducing ethnic inequalities in risk of STDR diagnosis.

## Methods

### Setting

The NELDESP is one of the largest, most ethnically diverse NHS screening programmes in the UK, with the neighbouring SELDESP also being a large ethnically diverse screening programme. The study used data from the NELDESP and SELDESP, collected from 3 January 2012 to 29 September 2023, with the data from the NELDESP also being linked with EHR. The study included anyone who could have been eligible for the biennial screening pathway, i.e. individuals with two consecutive screening appointments with a final outcome of no detectable retinopathy or maculopathy (R0 M0), in accordance with national definitions [25, 26], and at least one more gradable follow-up appointment. For screening visits with an ‘ungradable encounter’ (U) outcome, the next appointment with a gradable outcome was used instead of the appointment with an ungradable outcome, with appointments categorised as U not being included in the reported total number of appointments. People were censored when they would have left the biennial pathway due to evidence of any degree of diabetic retinopathy. Participants were included regardless of how reliably they attended screening appointments.

### Statistical analysis

All analyses were undertaken in R version 4.3.1 [27]. The primary health outcome was an STDR event, with STDR being defined as having R2 (pre-proliferative diabetic retinopathy), R3 (proliferative diabetic retinopathy) or M1 (diabetic maculopathy) in either eye [25]. The biennial protocol was simulated by ‘hiding’ alternate appointments that occurred less than 2 years after the previous appointment. As per the protocol, if an individual had an outcome of background diabetic retinopathy without maculopathy (R1 M0) [22], they returned to annual screening. If they had an outcome of R2, R3 or M1 on a ‘hidden’ appointment, they were considered to have had a delayed STDR diagnosis, while if they had an outcome of R2, R3 or M1 on a ‘seen’ appointment, their diagnosis was considered not to be delayed. The risk of being diagnosed with STDR at each appointment if appointments were scheduled biennially, instead of annually, was calculated, and the number of delayed diagnoses had the intervening annual appointments not taken place was estimated. Under the annual protocol, some individuals only attended their screening appointments every 2 years, in which case all their appointments were counted as they occurred and none of their diagnoses were considered delayed.

Variables available to the NELDESP included: self-described ethnic group (six categories comprising white, black, South Asian, any other Asian, mixed and other); age at registration (in years); age at screening appointment (in years); gender; smoking status (current, ex-smoker, never smoked, non-smoker but history unknown [reported as current non-smoker, but past smoking status unknown], vaping, unknown [both past and present status unknown]); diabetes type; date of diabetes diagnosis; and index of multiple deprivation (IMD) decile (derived from postcode, and reduced to quintiles) [28]; alongside diabetic retinopathy grading data and attendance record. Ethnicity data were collected using the nationally approved OptoMize eye screening software (NEC Software Solutions UK Limited), which uses ‘Ethnic Category Codes 2001’ as defined in the NHS Model and Dictionary [29]. These were then amalgamated into the six groups listed above. Similar data were available from the SELDESP for replication. Additional NELDESP variables from linked EHR included HbA_1c_ values, diastolic and systolic BP, and more accurate smoking status. The median HbA_1c_ value and the SD of HbA_1c_ values were calculated for each individual. For appointments with no (1.8%) or only one (3.6%) HbA_1c_ value recorded before the appointment, the missing median HbA_1c_ values and SD of HbA_1c_ values were imputed using the median of observed values (51 mmol/mol [6.8%] for the median and 6.2 mmol/mol [0.57%] for the SD). Similarly, for appointments with missing diastolic BP data (0.1%), the values were imputed using the median value of 76 mmHg, and for appointments with missing systolic BP data (0.1%), the values were imputed using the median value of 130 mmHg.

A mixed-effect logistic model was fitted, which was mutually adjusted for all covariates. Covariates that were significant, with a *p* value <0.05, were included in the prediction models, together with variables that were deemed clinically relevant. Further factors related to diabetic retinopathy progression were identified by analysing Kaplan–Meier plots and observing the mean risk of STDR diagnosis per annual appointment in the stratified groups (i.e. those stratified by age, ethnicity, diabetes duration). Fractional polynomials and backwards selection were used to select appropriate transformations for continuous variables.

A group of individuals in the simulated biennial screening protocol, with demographic characteristics that were associated with the lowest risk of delayed diagnosis of STDR, were chosen as a ‘base group’. We then attempted to identify a protocol that would triage people with diabetes into either annual or biennial screening, with the aim that all those in the biennial screening group would have a risk of a delayed diagnosis of STDR as similar as possible to that of the base group.

Mixed-effect logistic prediction models were created using all the information present at appointments with an R0 M0 outcome that directly followed at least one previous appointment with an R0 M0 outcome. The binary outcome was an STDR diagnosis in the next 590 days (i.e. within 2 years of the last screening appointment), with the person identifier being fitted as a random effect and all other covariates being fitted as fixed effects. Using these prediction models, the probability of STDR within 2 years was calculated at each subsequent appointment and if the probability was greater than the threshold, then their next appointment was considered ‘seen’ whenever it occurred, whilst if the probability was less than the threshold and within the next 730 days, the next appointment was considered ‘hidden’; if that appointment occurred after 730 days, it was considered ‘seen’. The threshold was set such that individuals in the highest 20% of STDR risk at a given appointment were considered ‘seen’. The simulation quantified how much better the protocols could be expected to perform in terms of relative risk of being diagnosed with STDR and reduction in number of appointments.

The optimal model was simplified into a points-based protocol by calculating the coefficients in the logistic regression for each year of age, for each year of diabetes duration and for ethnicity, and then rounding them to the nearest integer. Points were calculated for each participant by adding up the sum of the scores associated with their individual characteristics (see Text box). The threshold for requiring an annual screening appointment was set at the highest 20% of scores.


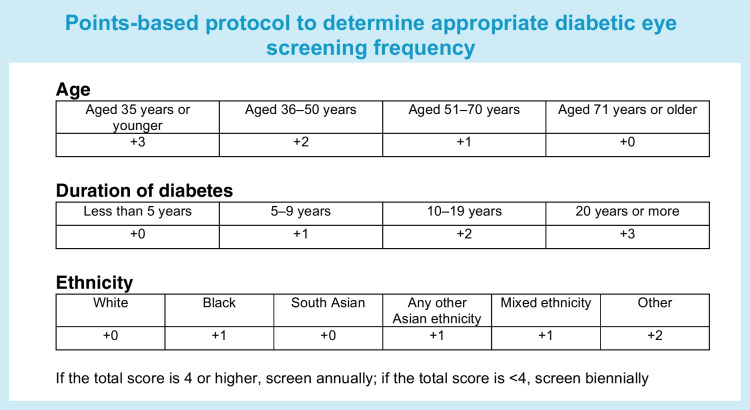


## Results

**Fig. 1:**
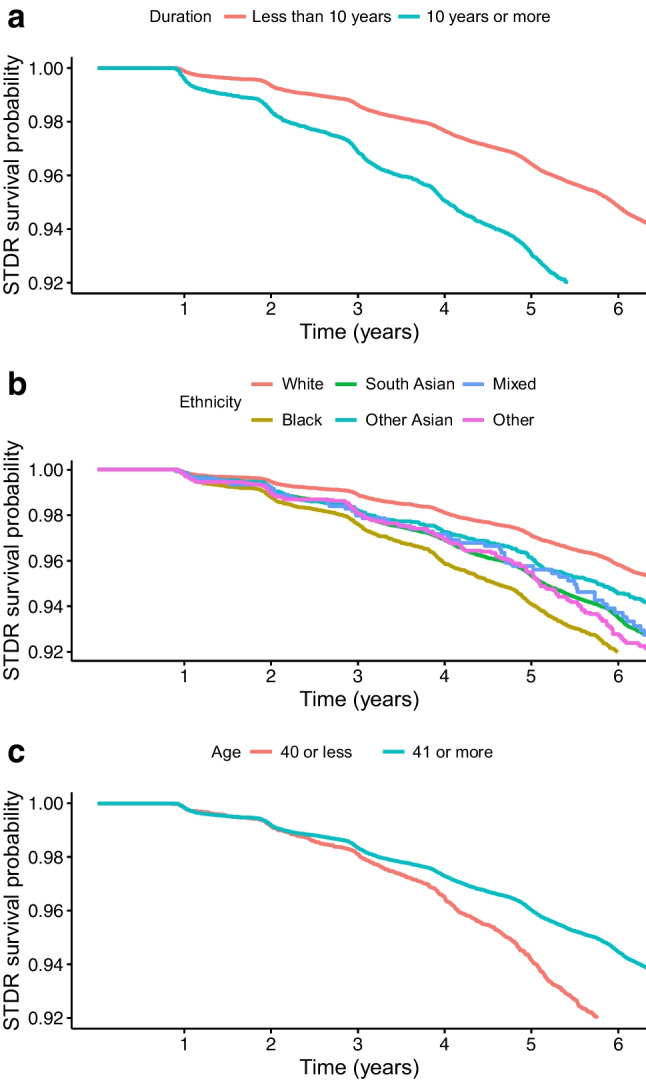
Kaplan–Meier survival plots for time until STDR diagnosis by (**a**) duration of diabetes (at start of biennial pathway), (**b**) ethnicity and (**c**) age (at start of biennial pathway)

Of the 200,626 people seen over 12 years in the NELDESP, 105,083 individuals met the inclusion criteria (see electronic supplementary material [ESM] Fig. 1). ESM Table 1 shows the baseline characteristics for the people included. The standard of care at the time of data collection was to invite individuals for screening at least once a year, resulting in 432,312 appointments and 3694 cases of STDR. The number of appointments per individual was 4.19, and there was a median duration of 402 days between the appointment in which STDR was diagnosed and the previous appointment. For every appointment, 854 individuals were diagnosed with STDR per 100,000 people screened, equivalent to a mean 0.9% risk of being diagnosed with STDR.

As shown in Table 1, the significant multivariable logistic regression covariates for incident STDR within 2 years of the previous screening appointment were age, IMD, prior non-attendance at screening appointment, and median HbA_1c_ value. Being an ex-smoker was associated with a higher risk of STDR compared with never having smoked; however, smoking status was not included in the final risk prediction models due to the high proportion of missing data and concerns about accuracy. The type of diabetes was included in models due to the different causes of type 1 and type 2 diabetes. In addition to absolute HbA_1c_ values, the SD of HbA_1c_ levels was also included in risk prediction models, as the variation in HbA_1c_ is considered clinically important [30]. People with a duration of diabetes of 10 years or longer had a higher risk of STDR compared with people with shorter durations (Fig. 1a); people of white ethnicity had a lower risk of STDR compared with the other ethnic groups (Fig. 1b); and people aged 40 years or younger were particularly at risk of STDR beyond 3 years from baseline compared with people who were 41 years or older (Fig. 1c). The cohort was therefore stratified by ethnicity, diabetes duration <10 years vs ≥10 years, and by age ≤40 years vs >40 years. People aged 40 years or younger were stratified by ethnicity only and not duration of diabetes due to the small sample size.

**Table 1:** Odds ratios showing factors related to STDR from multivariate logistic regression analyses

Characteristic	OR (95% CI)	*p* value
Gender		
Male (reference)		
Female	1.647 (0.5386, 5.0367)	0.38
Age category (years)		
<31	0.0042 (3 × 10^−4^, 0.0575)	4.3 × 10^−5^ ***
31 to 40	0.1288 (0.0529, 0.3134)	6.2 × 10^−6^ ***
41 to 50	0.3842 (0.2426, 0.6086)	4.6 × 10^−5^ ***
51 to 60 (reference)		
61 to 70	0.3882 (0.2381, 0.6332)	0.00015 ***
71 and over	0.5823 (0.2794, 1.2137)	0.15
Ethnicity		
White (reference)		
Black	1.0573 (0.206, 5.4264)	0.95
South Asian	3.2402 (0.7593, 13.8274)	0.11
Any other Asian	0.912 (0.0819, 10.1582)	0.94
Mixed	2.5991 (0.0169, 399.1855)	0.71
Other	2.7252 (0.0807, 92.0086)	0.58
Diabetes type		
Type 2 or other (reference)		
Type 1	2.6191 (0.1195, 57.4205)	0.54
Time since diagnosis		
Per year	1.0411 (0.9955, 1.0888)	0.078
IMD quintile^a^		
1 (reference)		
2	0.6351 (0.3198, 1.2611)	0.19
3	0.7015 (0.3086, 1.5946)	0.4
4	0.8299 (0.2356, 2.9232)	0.77
5	0.0019 (1 × 10^−4^, 0.0699)	0.00067 ***
Not given	0.3152 (0.0575, 1.7282)	0.18
BP (per mmHg)		
Diastolic	0.9734 (0.9082, 1.0432)	0.45
Systolic	1.0426 (0.9998, 1.0872)	0.051
HbA_1c_		
Median (mmol/mol)	1.0539 (1.0162, 1.0931)	0.0048 **
Per SD (mmol/mol)	1.0004 (0.9350, 1.0704)	0.99
Attendance record		
Previous non-attendance^b^	3.1822 (2.1444, 4.7221)	9 × 10^−9^ ***
Smoking status		
Never smoked (reference)		
Current smoker	0.5992 (0.2461, 1.4585)	0.26
Ex-smoker	2.4636 (1.1102, 5.4668)	0.027*
Non-smoker, history unknown	7.2445 (2 × 10^−4^, 237308.531)	0.71
Unknown	6.1389 (0.0291, 1296.0283)	0.51

^a^IMD indicates index of multiple deprivation [29]

^b^This is a binary variable indicating whether the person has previously not attended an appointment prior to their most recent appointment

Asterisks indicate statistically significant differences compared with the reference group: *p<0.05; **p<0.01; ***p<0.001

Four mixed-effect logistic regression models were derived, and the coefficients are shown in ESM Tables 2–5. Model 1 included the covariates available to both DESPs (age, diabetes duration, diabetes type and attendance record [ESM Table 2]), with model 2 also including ethnicity (ESM Table 3). Model 3 included the model 1 covariates as well as median HbA_1c_ value, the SD of HbA_1c_ and diastolic BP (ESM Table 4), with model 4 including all of the covariates included in model 3 plus ethnicity (ESM Table 5).

As shown in Table 2, for the biennial protocol, there would have been 199,776 appointments, equating to a reduction of 54% compared with the annual protocol. Out of the 3694 STDR events, although 2250 (61%) would not have had a delayed diagnosis, 1444 (39%) would have had a delayed diagnosis, which may have potentially resulted in delayed treatment and an increased risk of vision loss. These results mean that for every appointment under the biennial protocol, 1126 per 100,000 people screened were diagnosed with STDR (equating to a 1.1% risk of being diagnosed with STDR).

**Table 2:** Number of appointments and number of delayed STDR if the various screening protocols had been implemented in the NELDESP

	Annual	Biennial	Model 1	Model 2	Model 3	Model 4	Model 5
Number of appointments	432,312 (100%)	199,776 (46%)	237,755 (55%)	238,021 (55%)	238,738 (55%)	229,142 (53%)	245,270 (57%)
Number of non-delayed STDR	3694 (100%)	2250 (61%)	2515 (68%)	2700 (73%)	2554 (69%)	2297 (62%)	2761 (75%)

Model 1 included covariates available to the NELDESP (age, diabetes duration, whether diabetes was type 1 and attendance record). Model 2 also includes ethnicity. Model 3 includes the model 1 covariates as well as median HbA_1c_ value, the SD of HbA_1c_ and diastolic BP. Model 4 includes all of the above and ethnicity. Model 5 is a points-based risk score

Under the biennial protocol, the group with the lowest probability of an STDR diagnosis were people of white ethnicity, aged 41 years or older and who had lived with diabetes for less than 10 years (ESM Table 6). Hence, these individuals were chosen as the base group. Under the biennial protocol, the base group had a 1% risk of being diagnosed with STDR per appointment. In comparison, under the annual screening protocol, individuals of black ethnicity had a similar risk of being diagnosed with STDR per appointment (0.8%), but this risk was increased by threefold when the screening occurred biennially (2.4%) (ESM Table 6).

Table 3 shows the relative risk of being diagnosed with STDR at each appointment of the biennial protocol, and how it varies compared with the base group (raw data for probability of STDR diagnosis per appointment are given in ESM Table 6). Under the biennial protocol, all groups had a higher risk of STDR compared with the base group, and this risk was statistically significant at the 5% level in all but one subgroup (those aged 41 years or older, with a duration of diabetes of >10 years, and of mixed ethnicity). The number of screening appointments required and number of non-delayed STDR diagnoses are shown in Table 2. These data indicate that use of a protocol based on model 1 to triage individuals to annual vs biennial follow-up reduces the percentage of delayed STDR diagnoses to 32% of the annual screening protocol. However, use of this protocol also increases the number of appointments relative to the biennial protocol (55% vs 46% of the number of appointments in the annual protocol). Using model 3, the number of appointments was approximately the same as for model 1, and the percentage of delayed diagnoses only decreased slightly to 31%. Hence, inclusion of HbA_1c_ and BP in the model had a limited impact on reducing the number of screening appointments while reducing delays in STDR diagnosis.

**Table 3:** Risk ratios (95% CI) of STDR diagnosis per appointment under the various protocols in the NELDESP

	Annual	Biennial	Model 1	Model 2	Model 3	Model 4	Model 5
Aged 41 years or older, less than 10 years duration							
White	1	1	1	1	1	1	1
Black	2.29 (2.05, 2.57)	2.27 (2.02, 2.54)	2.40 (2.14, 2.69)	2.01 (1.8, 2.26)	2.43 (2.17, 2.72)	2.39 (2.13, 2.67)	1.76 (1.57, 1.97)
South Asian	1.65 (1.48, 1.83)	1.61 (1.45, 1.78)	1.7 (1.53, 1.88)	1.53 (1.38, 1.7)	1.69 (1.52, 1.88)	1.69 (1.52, 1.87)	1.58 (1.43, 1.76)
Other Asian	1.54 (1.29, 1.83)	1.49 (1.25, 1.77)	1.52 (1.28, 1.80)	1.32 (1.11, 1.57)	1.53 (1.29, 1.82)	1.52 (1.28, 1.80)	1.13 (0.95, 1.34)
Mixed	2.14 (1.55, 2.95)	2.19 (1.59, 3.02)	2.38 (1.73, 3.28)	1.95 (1.41, 2.69)	2.4 (1.74, 3.31)	2.37 (1.72, 3.27)	1.81 (1.31, 2.49)
Other	1.49 (1.15, 1.91)	1.49 (1.16, 1.91)	1.55 (1.21, 2.00)	1.02 (0.79, 1.31)	1.53 (1.19, 1.97)	1.55 (1.20, 1.99)	0.81 (0.63, 1.05)
Aged 41 years or older, more than 10 years duration							
White	1.61 (1.37, 1.9)	1.58 (1.34, 1.85)	1.14 (0.97, 1.34)	1.25 (1.06, 1.47)	1.13 (0.96, 1.33)	1.24 (1.05, 1.46)	1.13 (0.98, 1.35)
Black	3.44 (2.93, 4.03)	3.28 (2.81, 3.84)	2.58 (2.21, 3.02)	1.94 (1.66, 2.28)	2.5 (2.13, 2.92)	2.78 (2.38, 3.25)	1.57 (1.34, 1.84)
South Asian	3.33 (2.94, 3.77)	3.12 (2.75, 3.53)	2.48 (2.19, 2.81)	2.02 (1.78, 2.29)	2.30 (2.03, 2.61)	2.69 (2.38, 3.05)	2.16 (1.91, 2.45)
Other Asian	2.90 (2.26, 3.72)	2.71 (2.12, 3.48)	2.02 (1.57, 2.59)	1.56 (1.22, 2.00)	1.95 (1.52, 2.50)	2.19 (1.71, 2.80)	1.30 (1.02, 1.67)
Mixed	1.55 (0.58, 4.14)	1.54 (0.58, 4.07)	1.22 (0.46, 3.23)	0.79 (0.30, 2.11)	1.2 (0.45, 3.19)	1.36 (0.51, 3.61)	0.71 (0.27, 1.9)
Other	3.5 (2.46, 4.99)	3.34 (2.36, 4.75)	2.61 (1.84, 3.71)	1.66 (1.17, 2.36)	2.44 (1.72, 3.47)	2.89 (2.04, 4.11)	1.62 (1.13, 2.31)
Aged 40 years or younger							
White	2.54 (2.08, 3.1)	2.64 (2.17, 3.23)	3.09 (2.53, 3.77)	1.82 (1.49, 2.22)	3.06 (2.51, 3.73)	3.02 (2.47, 3.68)	1.84 (1.51, 2.24)
Black	4.09 (3.32, 5.05)	4.10 (3.33, 5.04)	4.79 (3.89, 5.89)	2.67 (2.17, 3.29)	4.74 (3.85, 5.83)	4.68 (3.80, 5.76)	2.30 (1.87, 2.83)
South Asian	2.05 (1.76, 2.38)	2.05 (1.76, 2.38)	2.39 (2.05, 2.78)	1.52 (1.30, 1.76)	2.37 (2.04, 2.76)	2.34 (2.01, 2.72)	1.63 (1.40, 1.89)
Other Asian	2.42 (1.70, 3.45)	2.42 (1.70, 3.44)	2.83 (1.99, 4.02)	1.6 (1.12, 2.27)	2.81 (1.98, 3.99)	2.77 (1.95, 3.93)	1.37 (0.96, 1.95)
Mixed	3.1 (1.61, 5.96)	3.6 (1.89, 6.86)	4.21 (2.21, 8.03)	2.22 (1.16, 4.26)	4.19 (2.19, 7.99)	4.11 (2.16, 7.85)	1.89 (0.99, 3.63)
Other	3.17 (1.96, 5.12)	3.22 (2.00, 5.17)	3.76 (2.34, 6.04)	1.77 (1.09, 2.85)	3.73 (2.32, 5.99)	3.68 (2.29, 5.91)	1.54 (0.95, 2.48)

Model 1 included covariates available to the NELDESP (age, diabetes duration, whether diabetes was type 1 and attendance record). Model 2 also includes ethnicity. Model 3 includes the model 1 covariates as well as median HbA_1c_ value, the SD of HbA_1c_ and diastolic BP. Model 4 includes all of the above and ethnicity. Model 5 is a points-based risk score

Using model 2, the number of appointments was approximately the same as for model 1, but the percentage of delayed STDR diagnoses decreased further to 27% of the annual screening protocol, as compared with the biennial screening programme, with which delayed STDR diagnosis was 39% of the annual screening programme. Furthermore, using model 4 decreased the number of appointments to 53% of the annual screening programme, but the percentage of delayed STDR diagnoses only decreased to 38%, offering little improvement compared with the biennial protocol.

Alongside the total reduction in screening appointments and number of delayed STDR diagnoses, we also assessed whether use of the prediction models equalises the risk of STDR diagnosis in various cohort subgroups (see Table 3). It was found that model 2, which uses NELDESP data including ethnicity, performed the best when it came to reducing inequality between the cohort subgroups, while removing ethnicity but including HbA_1c_ and BP (model 3) was not as effective. Across all models, most groups still had a significantly higher probability of an STDR diagnosis compared with the base group.

Model 2 was developed into model 5, a simplified points protocol that uses participant age, diabetes duration and ethnicity, with coefficients rounded (see Text box for details of the protocol). The threshold was set at ≥4 points to be invited for screening annually and <4 points to be invited biennially. This resulted in a greater number of screening appointments than for any of the other models, but still led to a 43% reduction in appointments as compared with the annual protocol (Table 2), and very comparable performance in terms of equalising risk of STDR diagnosis by cohort subgroup. After annual screening, which was set at 0% delayed STDR diagnoses, use of model 5 led to the lowest percentage of delayed STDR diagnoses (25% of the annual screening protocol; Table 2). All cohort subgroups had a more equitable probability of STDR diagnosis compared with the base group when using model 5 as compared with models 1, 3 and 4, whilst use of model 5 also resulted in more equitable probabilities of STDR diagnosis in all subgroups other than South Asians as compared with model 2 (Table 3).

Only models 1, 2 and 5 were applied to the SELDESP data, as linkage to EHR was unavailable. There were 150,201 people in the SELDESP, of which 79,296 were included in the analysis. Their characteristics are shown in ESM Table 7. Those with missing ethnicity information (2.6%) were assigned to the most common ethnic group within SELDESP (white). Compared with annual screening, the biennial protocol was predicted to reduce the number of appointments by 53% and result in a delayed STDR diagnosis for 34% of people (ESM Table 8). Use of model 1 (which excluded ethnicity) and annual screening of people in the top 20% for risk of STDR diagnosis reduced the number of appointments by 44% in the SELDESP cohort, as compared with annual screening, and resulted in a delayed STDR diagnosis for 27% of people (ESM Table 8), which was akin to the findings in the NELDESP cohort (Table 2). Applying model 2 (which included ethnicity) led to a similar number of appointments as annual screening, with a reduction of 45% of appointments compared with annual screening; however, the percentage of delayed STDR diagnoses was reduced further, to 23% (ESM Table 8). Upon applying model 5, the number of appointments was similarly reduced by 44% as compared with the annual screening protocol, whilst the percentage of delayed STDR diagnoses was 22% as compared with the annual screening protocol (ESM Table 8).

As seen in ESM Table 9, the risk ratio of STDR diagnosis per appointment for all groups compared with the base group was more equitable for all cohort subgroups for model 2 and model 5, as compared with the biennial protocol. Models 2 and 5 were more equitable for all but two subgroups (people of white ethnicity aged 41 years or more with diabetes duration of 10 years or more and people of South Asian ethnicity aged 41 years or more with diabetes duration of 10 years or more), as compared with model 1, indicating that including self-described ethnicity as a covariate improved the performance of the models. The raw probabilities are shown in ESM Table 10.

## Discussion

We have taken historical routine diabetic eye screening data from a large and diverse English DESP, gathered at a time when annual screening was offered to all people with diabetes in England. We have used this to simulate the effect of biennial recall in those who did not have detectable diabetic retinopathy at two consecutive screening appointments (the recently introduced criterion for biennial recall in the English national DESP). We have shown that there are considerable sociodemographic disparities in the risk of a delayed diagnosis of STDR when selecting people for biennial screening solely on this basis (two consecutive screening appointments without diabetic retinopathy). Therefore, the recent implementation of biennial screening appointments within the English national DESP [31] does not fairly, or effectively, allocate screening appointments to maximise timely STDR diagnoses. In particular, it leaves people of some ethnicities at higher risk of delayed STDR diagnosis, and potentially permanent sight loss. Other countries have implemented extended diabetic eye screening intervals among people perceived to be at ‘low risk’ [13, 32], but the present study provides strong evidence that such approaches may not be equitable, particularly in sociodemographically diverse populations, and should be monitored so that mitigation strategies can be devised. One such mitigation strategy is proposed here, which equalises sociodemographic inequity in STDR risk (e.g. by ethnicity, age and gender).

Given the introduction of the biennial screening policy in England, we have developed a simple protocol to address inequity in terms of sight-threatening diabetes complications, based on age, time since diagnosis and self-described ethnic group. The protocol triages people deemed to be at ‘low risk’ based on their retinal grade, and allocates annual or biennial screening follow-up (model 5). This protocol reduced the percentage of delayed STDR diagnoses from 39% with blanket biennial screening to 25%, while maintaining a reduced number of screening appointments (a 54% reduction when using the biennial protocol compared with annual screening, and a 43% reduction when using the revised protocol).

Use of ethnicity in the model was key to reducing inequality; however, even without inclusion of ethnicity data, a reduction in delayed STDR events was still seen. There is debate about utilising ethnicity in health prediction models without good evidence of risk reduction, with a previous medical risk prediction model for kidney disease removing ethnicity [33]. However, there is evidence of disparity between STDR progression in ethnic groups, and these differences cannot be explained by inclusion of further medical covariates [16, 17, 20, 34]. In the present study, incorporating HbA_1c_ and diastolic BP was not an adequate substitute for inclusion of ethnicity. Moreover, there are arguments that support the inclusion of ethnicity when there is clinical evidence to do so [35–37]. Although the NELDESP had a remarkably low rate of missing ethnicity data, reports indicate that other DESPs have a higher rate of missing ethnicity values [38]. In the SELDESP, everyone with missing ethnicity data was assumed to be of white ethnicity, which is unlikely.

Previous models focused on deep learning on the images to predict appropriate intervals between screening appointments, which lacks transparency and would be difficult to implement quickly across DESPs [39], whereas, our score-based protocol (model 5) is transparent, with a non-complicated formula and uses data that DESPs have. Such a score may be easily calculated and used by clinicians to guide screening intervals among those deemed to be at ‘low risk’. Some of the protocols outlined in this study, like other prediction models, do require covariates that the screening services do not currently have routine access to, such as HbA_1c_ and BP, meaning that some of the models could not be implemented without data linkage.

The median and SD of HbA_1c_ values were used in this study since this is what DESPs could have access to through data linkage. However, this may not represent the full extent of variation in HbA_1c_ levels given that this ‘low risk’ group are likely to have better diabetic control. Hence, the addition of HbA_1c_ data obtained before or at the time of screening did not add much to the models, with little impact in reducing the number of potential screening appointments.

The strengths of this study include the large number of participants and extended follow-up duration, with the inclusion of over 180,000 people from two different DESPs, followed up over a 10 year period, and the sociodemographic diversity of the study population, particularly ethnic diversity. Furthermore, the external validation in a different but similarly ethnically diverse DESP (the SELDESP) shows that a simple protocol to equalise risk could work in different settings across the UK. Moreover, the data emanates from an area with high levels of deprivation, ensuring representation of this under-served group. Importantly, linkage to EHR data that are not routinely available to the various DESPs within the English national programme allowed the contribution of these measures to be quantified in the prediction of STDR. The outcome, STDR diagnosis, was very well recorded, with diabetic retinopathy classification in both DESPs being carried out by trained assessors according to a multilevel, internally and externally quality-assured grading protocol that meets national recommendations [25].

A limitation of this study was that the simulation of the various protocols does not account for how people might engage with these strategies in real life. For example, people may disengage from the service due to the longer time period between screening appointments, with individuals who are asked to attend once every 2 years possibly decreasing their attendance further. This needs to be investigated further through use of prospective data, focus group research or ideally in a randomised controlled trial. However, such studies would need to be of large scale to examine the effects in population subgroups with sufficient statistical power and, with the rapid developments in technology, particularly of automated retinal image analysis systems to assist in human grading of retinal images routinely obtained in DESPs [40], the studies may well be outdated before completion. Furthermore, certain groups may need to come more often than every year, potentially every 6 months, to achieve an equal risk of being diagnosed with an STDR at each appointment. However, data at scale are not currently available to analyse the effect of six-monthly appointments. Further validation in non-UK diabetic eye screening settings would be required before global application.

While the percentage of STDR diagnoses delayed by the introduction of the biennial screening policy is considered by the UK National Screening Committee to be low (<1% per year) [31], the absolute numbers within a national screening programme will be appreciable, with marked sociodemographic inequality (disproportionately affecting non-white ethnic groups) [16]. While delayed treatment for some individuals with STDR may not result in irreversible complications [31], people with more serious disease cannot have treatment delayed, as this would probably result in irrecoverable sight loss. More work is needed to model different disease trajectories and their cost implications.

The score-based protocol described here, which does not require access to other primary or secondary EHR data, could offer a straightforward readily implementable solution to ameliorate potential inequalities caused by the recent introduction (in October 2023) of biennial screening among people deemed to be at ‘low risk’ of STDR based on their diabetic retinopathy grades. This would release capacity within the NHS by reducing the number of appointments needed among people at low risk, while limiting amplification of sociodemographic inequalities in healthcare [41]. If EHR data linkage were available for all DESPs, more accurate prediction models could be developed in the future. However, the data linkage required is not currently in place to support this action. Alternative pathways, such as automated retinal image analysis systems, which have been implemented elsewhere (including Scotland and Portugal) [40], could provide a safe, cost-effective alternative to enable more frequent screening intervals to be maintained, but these have not yet been licensed for universal use within the UK DESPs [42].

### Conclusions

This paper simulates the impact that the biennial screening interval protocol is likely to have on timely identification of sight-threatening diabetic eye disease, using real-world data. It shows that the biennial protocol is not fair or equitable across all groups of people living with diabetes, so it would seem reasonable to put an amelioration strategy in place. We propose a simple, readily implementable strategy based on age, time since diabetes diagnosis and ethnicity that can reduce the number of delayed STDR diagnoses while increasing equity in the probability of being diagnosed with STDR for all individuals. Importantly, this strategy showed remarkable consistency in performance both in the large, multi-ethnic development dataset and in the separate validation dataset.

## Electronic supplementary material

Below is the link to the electronic supplementary material.

ESM (PDF 182 KB)

## Data Availability

The North-East London and South-East London Diabetic Eye Screening Programme data are not publicly available because of restrictions on data sharing. A fully anonymised dataset is available from the programme upon reasonable request.

## Appendix

**The Artificial Intelligence & Automated Retinal Image Analysis Systems (ARIAS) Research Group** John Anderson, Sarah Barman, Louis Bolter, Ryan Chambers, Lakshmi Chandrasekaran, Umar Chaudhry, Emily Y. Chew, Catherine Egan, Jiri Fajtl, Frederick L. Ferris, Aroon D. Hingorani, Aaron Y. Lee, Abraham Olvera-Barrios, Christopher G. Owen, Paolo Remagnino, Alicja R. Rudnicka, Royce Shakespeare, Reecha Sofat, Adnan Tufail, Alasdair N. Warwick, Charlotte Wahlich, Roshan Welikala, Kathryn Willis, Yue Wu.

## Abbreviations

DESP: Diabetic Eye Screening Programme
EHR: Electronic health records
NELDESP: North East London Diabetic Eye Screening Programme
R0 M0: No detectable retinopathy or maculopathy
SELDESP: South East London Diabetic Eye Screening Programme
STDR: Sight-threatening diabetic retinopathy
U: Ungradable encounter
