# Supplementary material for: A simple score-based strategy to improve equity of the UK biennial diabetic eye screening protocol among people deemed as low risk
Source: Diabetologia. 2025 Mar 12;68(6):1157–68. doi: 10.1007/s00125-025-06379-6 (PMC12069130; doi:10.1007/s00125-025-06379-6)
Supplement: Supplementary file 1 — ESM (PDF 182 KB) [file 125_2025_6379_MOESM1_ESM.pdf]

Supplementary material

ESM Figure 1: Inclusion criteria for analysis within NELDESP

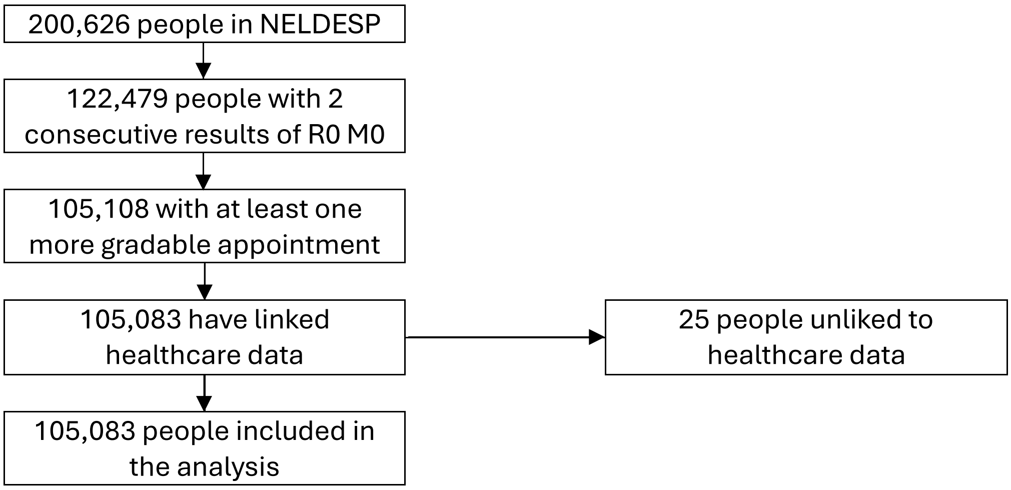

ESM Table 1: Baseline characteristics of the NEL DESP cohort among those with no DR on two consecutive annual screening visits and another gradable appointment

Characteristic	Number of people (%)
**Total**	105,083
**Age categories**	
<30yr	2976 (2.83%)
31 to 40yr	8468 (8.06%)
41 to 50yr	20549 (19.56%)
51 to 60yr	28234 (26.87%)
61 to 70yr	23676 (22.53%)
71yr and over	21180 (20.16%)
**Sex**	
Female	50152 (47.73%)
Male	54931 (52.27%)
**Ethnicity**	
White	38532 (36.67%)
Black	17170 (16.34%)
South Asian	38047 (36.21%)
Any other Asian	6922 (6.59%)
Mixed	1330 (1.27%)
Other	3082 (2.93%)
**Duration of diabetes**	
Less than 10 years	87540 (83.31%)
More than 10 years	17543 (16.69%)
**Type of diabetes**	
Type 2	100225 (95.38%)
Type 1	2947 (2.8%)
Other	58 (0.06%)
Missing	1853 (1.76%)
**Deprivation (IMD quintiles)**	
1	36365 (34.61%)
2	35129 (33.43%)
3	19393 (18.45%)
4	9388 (8.93%)
5	3434 (3.27%)
Not given	1374 (1.31%)

ESM Table 2: Logistic prediction Model 1 using DESP covariates and no ethnicity

Characteristic	Odds ratio (95% CI)	p-value	Coefficient
(Intercept)	7.64×10^{-13} (1×10^{-15}, 5.82×10^{-10})	1.69×10^{-16}	-27.9
Age in years	1.97 (1.72, 2.26)	4.52×10^{-22}	0.678
Age[a]	1.53e-23 (2.01×10^{-28}, 1.17×10^{-18})	5.35×10^{-20}	-52.5
Time since Diagnosis[b]	7170 (2020-25500)	7×10^{-43}	8.88
Type 1 diabetes	5.46×10^{-8} (4.96×10^{-10}, 6.01×10^{-6})	3.1×10^{-12}	-16.7
Time since diagnosis (days) /10000	0.313 (0.211, 0.464)	6.84×10^{-9}	-1.16
Previously not attended an appointment	0.0156 (0.00885, 0.0275)	5.11×10^{-47}	-4.16

[a]Age is the transformation of (age in years/100)^3

[b]Time since Diagnosis is the transformation of log(Time since diagnosis (days) /10000)

ESM Table 3: Logistic prediction Model 2 using DESP covariates and ethnicity

Characteristic	Odds ratio (95% CI)	p-value	Coefficient
(Intercept)	0.0188 (0.00163, 0.217)	0.00146	-3.97
Age in years	0.931 (0.885, 0.979)	0.0053	-0.0714
Age[a]	4.11 (0.0455, 371)	0.539	1.41
Time since Diagnosis[b]	4.48 (2.56, 7.84)	$1.5x10^{-7}$	1.5
Type 1 diabetes	0.658 (0.192, 2.25)	0.505	-0.419
Time since diagnosis (days) /10000	1.33 (0.279, 6.35)	0.72	0.285
Black	1.77 (0.936, 3.33)	0.0792	0.568
South Asian	1.07 (0.601, 1.92)	0.81	0.0711
Other Asian	1.7 (0.714, 4.04)	0.231	0.529
Mixed	2.13 (0.331, 13.7)	0.426	0.755
Other	4.2 (1.44, 12.3)	0.0088	1.44
Previously not attended an appointment	1.47 (1.05, 2.06)	0.0254	0.385

[a]Age is the transformation of (age in years/100)^3

[b]Time since Diagnosis is the transformation of log(Time since diagnosis (days) /10000)

ESM Table 4: Logistic prediction Model 3 using DESP and Health care record covariates, excluding ethnicity

Characteristic	Odds ratio (95% CI)	p-value	Coefficient
(Intercept)	5.55×10^{-6} (1.14×10^{-10}, 0.271)	0.028	-12.1
Age[a] in years	1.52 (1.35, 1.71)	2.95×10^{-12}	0.421
(age/100)^3	2.03×10^{-13} (1.75×10^{-17}, 2.36×10^{-09})	9.35×10^{-10}	-29.2
Time since Diagnosis[b]	8.83 (3.93, 19.9)	1.37×10^{-07}	2.18
Type 1 diabetes	7.03×10^{-6} (9.99×10^{-8}, 0.000494)	4.55×10^{-8}	-11.9
Time since diagnosis (days) /10000	1.43 (0.0766, 26.8)	0.81	0.36
Previously not attended an appointment	0.184 (0.117, 0.291)	3.58×10^{-13}	-1.69
Diastolic per mmHg	0.852 (0.795, 0.913)	6.1×10^{-6}	-0.16
Standard deviation of HbA1c mmol/mol values	0.9488 (0.7517, 1.1977)	0.658	-0.0526
HbA1c[c]	0.29 (0.0879, 0.957)	0.0422	-1.24
HbA1c[d]	21500 (612, 754000)	3.91×10^{-8}	9.98
HbA1c[e]	0.9942 (0.9886, 0.9998)	0.0413	-0.0058

[a]Age is the transformation of (age in years/100)^3

[b]Time since Diagnosis is the transformation of log(Time since diagnosis (days) /10000)

[c]HbA1c is the transformation of Median HbA1c mmol/mol / 10.929 +2.15

[d]HbA1c is the transformation of (Median HbA1c mmol/mol /109.29 +0.215)^3

[e]HbA1c is the transformation of (Standard deviation of HbA1c mmol/mol values) ^2

ESM Table 5: Logistic prediction Model 4 using DESP and health record covariates, including ethnicity

Characteristic	Odds ratio (95% CI)	p-value	Coefficient
(Intercept)	0.00012 ($3.52x10^{-5}$, 0.000412)	$7.16x10^{-47}$	-9.02
Age in years	0.963 (0.95, 0.977)	$1.26x10^{-7}$	-0.0377
Age[a]	19.5 (5.87, 65)	$1.26x10^{-6}$	2.97
Time since diagnosis (days) /10000	0.832 (0.474, 1.46)	0.523	-0.184
Time since Diagnosis[b]	1.74 (1.39, 2.17)	$9.81x10^{-7}$	0.552
Type 1 diabetes	0.786 (0.551, 1.12)	0.185	-0.24
Black	2.02 (1.74, 2.35)	$9.76x10^{-20}$	0.704
South Asian	1.47 (1.27, 1.69)	$1.27x10^{-07}$	0.383
Other Asian	1.62 (1.3, 2.01)	$1.35x10^{-5}$	0.481
Mixed	1.84 (1.15, 2.94)	0.0106	0.61
Other	1.15 (0.799, 1.64)	0.457	0.137
Standard deviation of HbA1c mmol/mol	1.05 (1.03, 1.07)	$6.64x10^{-7}$	0.0468
HbA1c[c]	816 (195, 3420)	$4.63x10^{-20}$	6.7
Previously not attended an appointment	1.27 (1.14, 1.41)	$1.44x10^{-5}$	0.238
diastolic	1.01 (1, 1.02)	0.000656	0.0111
HbA1c[d]	0.313 (0.18, 0.544)	$3.81x10^{-5}$	-1.16
HbA1c[e]	0.9990 (0.9985, 0.9994)	$8.85x10^{-6}$	-0.001

[a]Age is the transformation of (age in years/100)^3

[b]Time since Diagnosis is the transformation of log(Time since diagnosis (days) /10000)

[c]HbA1c is the transformation of Median HbA1c mmol/mol / 109.29 +0.215

[d]HbA1c is the transformation of (Median HbA1c mmol/mol /109.29 +0.215)^3

[e]HbA1c is the transformation of (Standard deviation of HbA1c values) ^2

ESM Table 6: Probability of STDR diagnosis per appointment in NEL DESP

	Annual	Biennial	Model 1 (DESP excluding ethnicity)	Model 2 (DESP including ethnicity)	Model 3 (Healthcare records excluding ethnicity)	Model 4 (Healthcare records including ethnicity)	Model 5 (Points risk Score)
**Aged 41 years or older, less than 10 years duration of diabetes**							
White	0.00374 (0.00345, 0.00405)	0.0104 (0.00962, 0.0113)	0.00891 (0.00822, 0.00966)	0.0103 (0.00947, 0.0111)	0.00896 (0.00827, 0.00971)	0.00911 (0.00841, 0.00988)	0.0104 (0.00956, 0.0112)
Black	0.00858 (0.00791, 0.00931)	0.0239 (0.022, 0.0259)	0.0216 (0.0199, 0.0234)	0.0208 (0.0192, 0.0225)	0.0219 (0.0202, 0.0238)	0.0219 (0.0202, 0.0238)	0.0183 (0.0169, 0.0198)
South Asian	0.00616 (0.00575, 0.00658)	0.0168 (0.0158, 0.018)	0.0152 (0.0142, 0.0163)	0.0158 (0.0148, 0.0169)	0.0152 (0.0142, 0.0163)	0.0155 (0.0145, 0.0166)	0.0165 (0.0154, 0.0176)
Other Asian	0.00575 (0.00493, 0.00671)	0.0156 (0.0134, 0.0181)	0.0136 (0.0117, 0.0158)	0.0136 (0.0117, 0.0158)	0.0138 (0.0118, 0.016)	0.0139 (0.0119, 0.0162)	0.0117 (0.0101, 0.0137)
Mixed	0.00799 (0.00585, 0.0109)	0.023 (0.0169, 0.0314)	0.0214 (0.0157, 0.0291)	0.0201 (0.0147, 0.0274)	0.0216 (0.0159, 0.0294)	0.0218 (0.016, 0.0297)	0.0188 (0.0138, 0.0256)
Other	0.00556 (0.00438, 0.00705)	0.0156 (0.0123, 0.0197)	0.0139 (0.011, 0.0176)	0.0105 (0.00827, 0.0133)	0.0138 (0.0109, 0.0175)	0.0142 (0.0112, 0.0179)	0.00845 (0.00666, 0.0107)
**Aged 41 years or older, 10 years duration of diabetes or more**							
White	0.00603 (0.00523, 0.00695)	0.0166 (0.0144, 0.0191)	0.0101 (0.00881, 0.0117)	0.0129 (0.0112, 0.0149)	0.0101 (0.00877, 0.0116)	0.0113 (0.00984, 0.0131)	0.012 (0.0104, 0.0138)
Black	0.0129 (0.0112, 0.0147)	0.0348 (0.0304, 0.0397)	0.0232 (0.0202, 0.0265)	0.02 (0.0175, 0.0229)	0.0225 (0.0196, 0.0257)	0.0256 (0.0224, 0.0293)	0.0163 (0.0142, 0.0186)
South Asian	0.0124 (0.0113, 0.0137)	0.033 (0.03, 0.0362)	0.0223 (0.0202, 0.0245)	0.0208 (0.0189, 0.0229)	0.0207 (0.0188, 0.0228)	0.0248 (0.0225, 0.0272)	0.0225 (0.0205, 0.0248)
Other Asian	0.0109 (0.00857, 0.0137)	0.0286 (0.0227, 0.0362)	0.0181 (0.0143, 0.0229)	0.0161 (0.0127, 0.0203)	0.0176 (0.0139, 0.0222)	0.0201 (0.0159, 0.0254)	0.0135 (0.0107, 0.0171)
Mixed	0.00581 (0.00226, 0.0148)	0.0162 (0.00632, 0.0409)	0.0109 (0.00425, 0.0277)	0.00815 (0.00317, 0.0208)	0.0108 (0.00421, 0.0275)	0.0125 (0.00487, 0.0317)	0.00738 (0.00287, 0.0188)
Other	0.0131 (0.00929, 0.0184)	0.0353 (0.0251, 0.0494)	0.0234 (0.0166, 0.0329)	0.017 (0.0121, 0.0239)	0.022 (0.0156, 0.0309)	0.0268 (0.019, 0.0376)	0.0168 (0.0119, 0.0236)
**Aged 40 years or younger**							
White	0.00949 (0.0079, 0.0114)	0.0278 (0.0232, 0.0333)	0.0277 (0.0231, 0.0332)	0.0187 (0.0156, 0.0224)	0.0276 (0.023, 0.0331)	0.0277 (0.0231, 0.0332)	0.0191 (0.0159, 0.0229)

Black	0.0153 (0.0126, 0.0186)	0.0435 (0.0359, 0.0525)	0.0434 (0.0358, 0.0525)	0.0275 (0.0227, 0.0333)	0.0432 (0.0357, 0.0522)	0.0434 (0.0358, 0.0525)	0.0239 (0.0197, 0.0289)
South Asian	0.00766 (0.00674, 0.00871)	0.0215 (0.0189, 0.0244)	0.0214 (0.0188, 0.0243)	0.0156 (0.0137, 0.0177)	0.0214 (0.0188, 0.0243)	0.0214 (0.0189, 0.0243)	0.0169 (0.0149, 0.0192)
Other Asian	0.00906 (0.00643, 0.0128)	0.0254 (0.018, 0.0356)	0.0254 (0.018, 0.0356)	0.0164 (0.0116, 0.0231)	0.0253 (0.018, 0.0355)	0.0254 (0.018, 0.0356)	0.0142 (0.0101, 0.02)
Mixed	0.0116 (0.00611, 0.0219)	0.0378 (0.02, 0.0703)	0.0378 (0.02, 0.0703)	0.0228 (0.012, 0.0427)	0.0378 (0.02, 0.0703)	0.0378 (0.02, 0.0703)	0.0196 (0.0103, 0.0368)
Other	0.0119 (0.00742, 0.0189)	0.0339 (0.0213, 0.0537)	0.0339 (0.0213, 0.0537)	0.0181 (0.0113, 0.0288)	0.0338 (0.0212, 0.0535)	0.0339 (0.0213, 0.0537)	0.0159 (0.00997, 0.0254)

ESM Table 7: Baseline characteristics of the SELDESP cohort among those with no DR on two consecutive annual screening visits and another gradable appointment

Characteristic	Number of people (%)
**Total**	79296
**Age categories**	
<31yr	2162 (2.7%)
31 to 40yr	4106 (5.2%)
41 to 50yr	12450 (15.7%)
51 to 60yr	21742 (27.1%)
61 to 70yr	19694 (24.8%)
71yr and over	19412 (24.5%)
**Sex**	
Female	37371 (47.1%)
Male	41910 (52.9%)
Missing	15 (0.02%)
**Ethnicity**	
White	41335 (52.1%)
Black	21471 (27.1%)
South Asian	5202 (6.6%)
Any other Asian	5054 (6.4%)
Mixed	2103 (2.7%)
Other	2035 (2.6%)
Missing	2096 (2.6%)
**Duration of diabetes**	
Less than 10 years	64845(81.8%)
More than 10 years	14451 (18.2%)
**Type of diabetes**	
Type 2	75943 (95.8%)
Type 1	3017 (3.8%)
Other	121 (0.15%)
Missing	215 (0.27%)
**Deprivation (IMD quintiles)**	
1	9900 (12.5%)
2	8746(11.0%)
3	4669 (5.9%)
4	2616 (3.3%)
5	1789 (2.3%)
Not given	51576 (65.0%)

ESM Table 8: Number of appointments and number of delayed STDR if the screening protocols had been implemented in SEL DESP

	Annual	Biennial	Model 1[a] (DESP excluding ethnicity)	Model 2[a] (DESP including ethnicity)	Model 5[a] (Points risk Score)
Number of appointments	295,805 (100%)	140,131 (47%)	165,768 (56%)	162,781(55%)	166,469 (56%)
Number of non-delayed STDR	2532 (100%)	1662 (66%)	1858 (73%)	1949 (77%)	1963 (78%)

[a] The models were applied to all appointments and those with the highest 20% of risk were seen annually with everyone else seen biennially

ESM Table 9: Risk Ratio (95% CI) of STDR diagnosis per appointment under different protocols in SEL DESP

	Annual	Biennial	Model 1 (DESP excluding ethnicity)	Model 2 (DESP including ethnicity)	Model 5 (Points risk Score)
**Aged 41 years or older, less than 10 years duration of diabetes**					
White	1 (1, 1)	1 (1, 1)	1 (1, 1)	1 (1, 1)	1 (1, 1)
Black	2.33 (2.09, 2.59)	2.36 (2.13, 2.63)	2.43 (2.18, 2.70)	2.13 (1.91, 2.37)	1.96 (1.76, 2.18)
South Asian	1.47 (1.22, 1.76)	1.6 (1.33, 1.91)	1.58 (1.32, 1.89)	1.53 (1.28, 1.83)	1.56 (1.3, 1.87)
Other Asian	1.39 (1.16, 1.68)	1.53 (1.27, 1.83)	1.52 (1.26, 1.82)	1.38 (1.15, 1.66)	1.27 (1.06, 1.53)
Mixed	1.71 (1.38, 2.10)	2.03 (1.65, 2.5)	1.98 (1.61, 2.44)	1.81 (1.47, 2.23)	1.78 (1.44, 2.19)
Other	1.50 (1.20, 1.88)	1.8 (1.45, 2.25)	1.76 (1.41, 2.20)	1.45 (1.16, 1.81)	1.29 (1.03, 1.61)
**Aged 41 years or older, 10 years duration of diabetes or more**					
White	1.64 (1.43, 1.9)	1.68 (1.46, 1.94)	1.14 (0.99, 1.31)	1.43 (1.24, 1.64)	1.52 (1.32, 1.75)
Black	3.57 (3.12, 4.07)	3.8 (3.33, 4.33)	2.68 (2.35, 3.06)	2.56 (2.24, 2.92)	2.46 (2.15, 2.8)
South Asian	2.35 (1.93, 2.85)	2.79 (2.30, 3.37)	2.11 (1.74, 2.56)	2.29 (1.89, 2.78)	2.51 (2.07, 3.04)
Other Asian	1.77 (1.41, 2.22)	2.15 (1.72, 2.69)	1.65 (1.32, 2.07)	1.63 (1.30, 2.04)	1.58 (1.26, 1.98)
Mixed	1.89 (1.48, 2.41)	2.5 (1.97, 3.19)	2.13 (1.67, 2.71)	2.07 (1.62, 2.63)	2.12 (1.66, 2.7)
Other	1.78 (1.38, 2.28)	2.36 (1.84, 3.03)	1.98 (1.54, 2.55)	1.87 (1.46, 2.40)	1.89 (1.47, 2.42)
**Aged 40 years or younger**					
White	2.42 (2.05, 2.85)	2.85 (2.42, 3.36)	2.91 (2.47, 3.43)	2.14 (1.81, 2.52)	2.13 (1.81, 2.51)
Black	3.17 (2.70, 3.72)	3.74 (3.20, 4.39)	3.77 (3.21, 4.41)	2.82 (2.41, 3.31)	2.58 (2.2, 3.02)
South Asian	1.95 (1.55, 2.45)	2.53 (2.02, 3.18)	2.44 (1.94, 3.06)	2.17 (1.73, 2.72)	2.25 (1.79, 2.82)
Other Asian	1.97 (1.56, 2.48)	2.56 (2.04, 3.22)	2.46 (1.96, 3.09)	2.11 (1.68, 2.66)	2.02 (1.61, 2.54)
Mixed	1.77 (1.37, 2.28)	2.4 (1.86, 3.09)	2.26 (1.75, 2.91)	2.05 (1.59, 2.64)	2.04 (1.58, 2.63)
Other	1.68 (1.29, 2.19)	2.29 (1.76, 2.98)	2.14 (1.65, 2.79)	1.96 (1.51, 2.55)	1.95 (1.5, 2.53)

ESM Table 10: Probability of STDR diagnosis per appointment in SEL DESP

	Annual	Biennial	Model 1 (DESP excluding ethnicity)	Model 2 (DESP including ethnicity)	Model 5 (Points risk Score)
**Aged 41 years or older, less than 10 years duration of diabetes**					
White	0.00351 (0.00323,0.00381)	0.00968 (0.00891,0.0105)	0.00888 (0.00817,0.00965)	0.00955 (0.00879,0.0104)	0.00962 (0.00886,0.0105)
Black	0.00876 (0.00813,0.00943)	0.0244 (0.0227,0.0262)	0.0231 (0.0215,0.0248)	0.0215 (0.02,0.0231)	0.0198 (0.0184,0.0213)
South Asian	0.00498 (0.00404,0.00613)	0.0137 (0.0111,0.0169)	0.0127 (0.0104,0.0157)	0.0131 (0.0106,0.0161)	0.0135 (0.011,0.0166)
Other Asian	0.0046 (0.00372,0.0057)	0.0128 (0.0103,0.0158)	0.0119 (0.00966,0.0148)	0.0112 (0.00909,0.0139)	0.0102 (0.00822,0.0126)
Mixed	0.0064 (0.00482,0.0085)	0.018 (0.0135,0.0238)	0.017 (0.0128,0.0225)	0.0153 (0.0115,0.0203)	0.0147 (0.0111,0.0195)
Other	0.00476 (0.00342,0.00661)	0.0136 (0.00981,0.0189)	0.0129 (0.00928,0.0179)	0.00964 (0.00694,0.0134)	0.00794 (0.00571,0.011)
**Aged 41 years or older, 10 years duration of diabetes or more**					
White	0.00598 (0.00524,0.00682)	0.0161 (0.0142,0.0184)	0.00955 (0.00837,0.0109)	0.0133 (0.0116,0.0151)	0.0144 (0.0126,0.0164)
Black	0.0166 (0.0148,0.0187)	0.0448 (0.0399,0.0502)	0.0264 (0.0235,0.0296)	0.0267 (0.0238,0.03)	0.0255 (0.0227,0.0286)
South Asian	0.0127 (0.01,0.0161)	0.0337 (0.0267,0.0425)	0.0195 (0.0154,0.0246)	0.0241 (0.0191,0.0305)	0.0285 (0.0226,0.036)
Other Asian	0.00722 (0.00509,0.0102)	0.0192 (0.0136,0.0272)	0.011 (0.00774,0.0155)	0.0114 (0.00806,0.0162)	0.0107 (0.00754,0.0151)
Mixed	0.0116 (0.00735,0.0183)	0.0319 (0.0203,0.0499)	0.0191 (0.0121,0.03)	0.0181 (0.0115,0.0284)	0.0187 (0.0119,0.0294)
Other	0.00893 (0.00523,0.0152)	0.0243 (0.0143,0.0412)	0.0138 (0.00809,0.0235)	0.0118 (0.00693,0.0202)	0.0117 (0.00686,0.02)
**Aged 40 years or younger**					
White	0.0109 (0.00918,0.013)	0.0323 (0.0272,0.0383)	0.0322 (0.0271,0.0382)	0.0214 (0.018,0.0254)	0.0212 (0.0179,0.0252)
Black	0.0176 (0.015,0.0207)	0.0499 (0.0425,0.0585)	0.0496 (0.0422,0.0582)	0.0321 (0.0273,0.0377)	0.0279 (0.0237,0.0328)
South Asian	0.0105 (0.0073,0.015)	0.0304 (0.0212,0.0433)	0.0302 (0.0211,0.0431)	0.0217 (0.0151,0.031)	0.0232 (0.0162,0.0332)
Other Asian	0.0111 (0.00766,0.0159)	0.0319 (0.0222,0.0458)	0.0318 (0.0221,0.0456)	0.0201 (0.014,0.029)	0.0174 (0.0121,0.0251)
Mixed	0.00922 (0.00516,0.0164)	0.0274 (0.0153,0.0483)	0.0274 (0.0153,0.0483)	0.0161 (0.00902,0.0286)	0.0147 (0.00821,0.0261)
Other	0.00676 (0.0031,0.0147)	0.019 (0.00876,0.0409)	0.019 (0.00873,0.0408)	0.0105 (0.00481,0.0227)	0.00929 (0.00426,0.0201)
